# Association between eating disorders and anaphylaxis

**DOI:** 10.1186/2050-2974-3-S1-P2

**Published:** 2015-11-23

**Authors:** Leanne Barron

**Affiliations:** 1 QUT Eating Disorders Clinic/Brisbane City Doctors, Australia

## 

In a retrospective study of 113 patients presenting to a Brisbane general practitioner with eating disorders, 14 patients gave a history of anaphylaxis, whilst another 7 reported significant food allergies. This is significantly higher than population estimates of anaphylaxis of 1 in 1700.

This high incidence leads one to ponder the origins of the issues - whether the fear of anaphylaxis contributes to a fear of food in general (with implications for management of children with food allergies) or whether the two conditions may share underlying immunological and/or genetic risk factors.

Of particular note is the recent treatment of anaphylaxis in children using probiotics, the known association of PANDAS, recent discoveries linking the micro biome to mental health and the increased rate of asthma in mothers with depression.

